# Supplementary material for: Clinical Significance of Adenosine-Induced Atrial Fibrillation after Complete Pulmonary Vein Isolation
Source: J Clin Med. 2022 Sep 26;11(19):5679. doi: 10.3390/jcm11195679 (PMC9570534; doi:10.3390/jcm11195679)
Supplement: Supplementary file 1 [file jcm-11-05679-s001.zip › jcm-1836429-supplementary.pdf]

**Supplementary Table S1. Recurrence rate of trigger sites.**

| Trigger Site | Additional Ablation | Recurrence |
|---|---|---|
| Crista terminalis | 7 | 2 |
| Right atrial appendage | 2 | 0 |
| Vein of Marshall | 1 | 0 |

**Supplementary Figure S1. Adenosine induced atrial fibrillation after long atrioventricular block.**

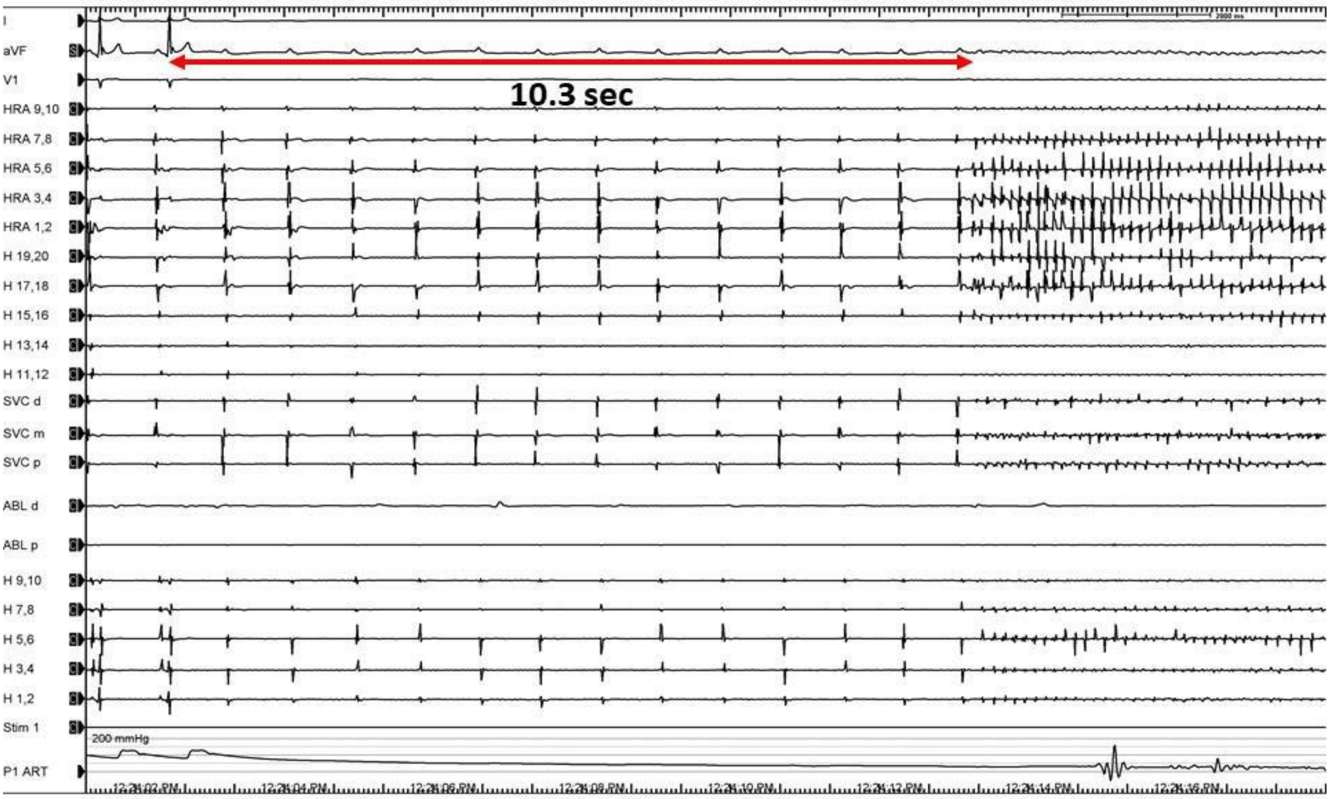


Adenosine testing was performed to confirm dormant conduction after pulmonary vein isolation. A circular mapping catheter was placed in the LSPV and adenosine was administered. Atrioventricular block occurred for more than 10 s, and adenosine-induced atrial fibrillation was observed.
